# Roles of sarcoplasmic reticulum Ca^2+^ ATPase pump in the impairments of lymphatic contractile activity in a metabolic syndrome rat model

**DOI:** 10.1038/s41598-020-69196-4

**Published:** 2020-07-23

**Authors:** Yang Lee, Sanjukta Chakraborty, Mariappan Muthuchamy

**Affiliations:** 1 Department of Medical Physiology, College of Medicine, Texas A&M University, Bryan, TX 77807 USA; 2 Present Address: Vascular Biology Program, Boston Children’s Hospital, Harvard Medical School, Boston, MA 02115 USA

**Keywords:** Cell biology, Molecular biology, Physiology

## Abstract

The intrinsic lymphatic contractile activity is necessary for proper lymph transport. Mesenteric lymphatic vessels from high-fructose diet-induced metabolic syndrome (MetSyn) rats exhibited impairments in its intrinsic phasic contractile activity; however, the molecular mechanisms responsible for the weaker lymphatic pumping activity in MetSyn conditions are unknown. Several metabolic disease models have shown that dysregulation of sarcoplasmic reticulum Ca^2+^ ATPase (SERCA) pump is one of the key determinants of the phenotypes seen in various muscle tissues. Hence, we hypothesized that a decrease in SERCA pump expression and/or activity in lymphatic muscle influences the diminished lymphatic vessel contractions in MetSyn animals. Results demonstrated that SERCA inhibitor, thapsigargin, significantly reduced lymphatic phasic contractile frequency and amplitude in control vessels, whereas, the reduced MetSyn lymphatic contractile activity was not further diminished by thapsigargin. While SERCA2a expression was significantly decreased in MetSyn lymphatic vessels, myosin light chain 20, MLC_20_ phosphorylation was increased in these vessels. Additionally, insulin resistant lymphatic muscle cells exhibited elevated intracellular calcium and decreased SERCA2a expression and activity. The SERCA activator, CDN 1163 partially restored lymphatic contractile activity in MetSyn lymphatic vessel by increasing phasic contractile frequency. Thus, our data provide the first evidence that SERCA2a modulates the lymphatic pumping activity by regulating phasic contractile amplitude and frequency, but not the lymphatic tone. Diminished lymphatic contractile activity in the vessels from the MetSyn animal is associated with the decreased SERCA2a expression and impaired SERCA2 activity in lymphatic muscle.

## Introduction

Insulin resistance is one of the major causes of metabolic syndrome (MetSyn) or related metabolic disorders that are associated with an enormous health burden worldwide^1^. MetSyn is now one of the most prevalent diseases globally and increases the risk for all causes of mortalities, including cardiovascular diseases^1,2^. Clinical studies have established the link between obesity and lymphatic dysfunction, which is associated with increased susceptibility for developing lymphedema^3–6^. Mice heterozygous for Prox1, a master lymphatic endothelial transcription factor, consistently develop adult onset obesity coupled with increased chyle accumulation in the thoracic cavity^7,8^. In addition, these mice exhibited higher leptin and insulin levels^8^, which are pathological determinant factors of insulin resistance suggesting a direct role of the lymphatic system in metabolic dysfunction. We have previously reported that a high-fructose-fed rat model of MetSyn presented a significant reduction in lymphatic pumping as a consequence of decreased phasic contractile frequency and impaired intrinsic lymphatic muscle force production^9,10^. These findings have been corroborated in the obese mouse models that diminished pressure-induced frequency in collecting lymphatic vessels^11^. We have also demonstrated that insulin resistance directly impaired cellular bioenergetics and decreased the relative levels of the regulatory molecule, myosin light chain 20 (MLC_20_) in lymphatic muscle cells (LMCs). However, the direct mechanisms that reduce lymphatic pumping activity in the MetSyn animals have not been completely understood.

The active spontaneous pumping of lymphatics is achieved by the intrinsic contractile activity of the lymphatic muscle cells in the wall of collecting lymphatic vessels that produces the rhythmic phasic contractions. The lymphatic muscle cells exhibit unique characteristics similar to both vascular smooth muscle and cardiac muscle cells^12^. Like vascular smooth muscle, lymphatics show the contractile activity that is regulated by various vasoactive (e.g., substance p, endothelin-1, histamine, acetylcholine, etc.) and mechanical factors (e.g., transmural pressure, flow, etc.). In addition, lymphatic muscle displays a rapid phasic contraction that is mainly achieved by the intrinsic pumping characteristics. While the resting membrane potential is mediated by Cl^−^^13^ and voltage gated K^+^ channel^14^, lymphatic contractions are predominantly regulated by Ca^2+^ influx^15,16^. The intracellular Ca^2+^ concentration determines the lymphatic vessel contraction and similar to most other smooth muscle types, Ca^2+^ binds to calmodulin to form an active Ca^2+^/calmodulin complex, which activates myosin light chain kinase, a key regulatory molecule that phosphorylate MLC_20_^17–20^. Since MLC_20_ phosphorylation is increased in the insulin resistant LMCs^21^, we propose that regulatory molecules of Ca^2+^ and/or Ca^2+^ homeostasis are impaired in the MetSyn lymphatics.

The endoplasmic reticulum (ER) is the main storage site of intracellular Ca^2+^ that maintains intracellular Ca^2+^ levels ~ 10,000-fold lower than extracellular and ER Ca^2+^ concentrations^22,23^. Re-uptake of Ca^2+^ into the ER by sarcoplasmic reticulum Ca^2+^-ATPase (SERCA) is necessary for muscle relaxation and restores ER Ca^2+^ levels for subsequent systolic and diastolic cycles followed by transiently increased intracellular Ca^2+^ levels. Alterations in Ca^2+^ homeostasis have been shown to trigger lymphatic dysfunction. When L-type Ca^2+^ channels were disrupted, stretch-induced lymphatic contractile amplitude was diminished; whereas, T-type, ‘transient,’ Ca^2+^ channel inhibition reduced the stretch-induced phasic contractile frequency in the lymphatics^24^. Disrupting ER Ca^2+^ in isolated bovine lymphatic vessels caused lymphatic dysfunction^25^. Additionally, SERCA2 activity and expression are diminished in vascular smooth muscle^26,27^ and heart^28,29^ in different animal models of obesity/diabetes, highlighting a potential pathological role for SERCA2 dysfunction and disturbed intracellular Ca^2+^ homeostasis in the development of metabolic abnormalities in insulin resistance and diabetes. However, the role of SERCA2 in lymphatic pumping activity and possible pathophysiological roles in MetSyn have not yet been examined.

Our previous data showed negative chronotropic effects at all transmural pressures that effectively reduced the intrinsic flow generating capacity of mesenteric lymphatic vessels in MetSyn rats^9,10^. Additionally, insulin resistance increased MLC_20_ phosphorylation in LMCs^21^ that is mediated by intracellular Ca^2+^^20^. Hence, we hypothesized that a decrease in SERCA expression and/or activity in lymphatic muscle influences Ca^2+^ homeostasis in LMCs, and consequently, diminishes lymphatic contractile activity in MetSyn animals. To test this hypothesis, we assessed the expression of SERCA2 isoforms in lymphatic muscle and determined the role of SERCA2 in the regulation of lymphatic contraction in the normal and MetSyn conditions.

## Results

### MetSyn rats exhibit an altered body composition by decreasing skeletal muscle mass, but increasing body fat deposition and cardiac muscle mass

**Figure 1:**
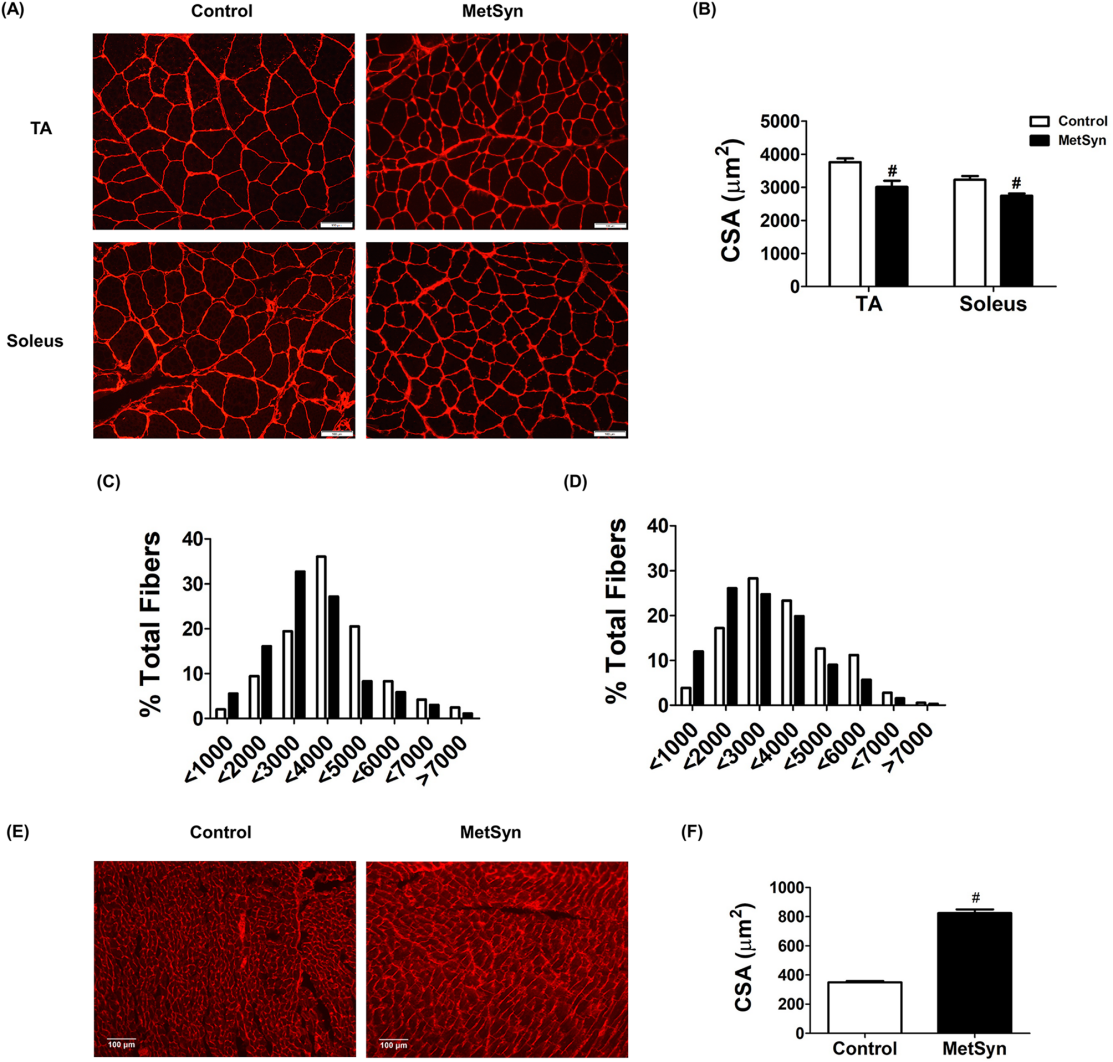
Skeletal muscle atrophy and cardiac hypertrophy in MetSyn animals. (**A**) WGA staining shows skeletal muscle sarcolemma staining in tibialis anterior, TA (upper panel) and Soleus muscles (lower panel) for control (left) and MetSyn (right). Image were obtained with × 20 objective (NA = 0.7) on a fluorescence microscope. (**B**) Cross sectional area, CSA of TA and soleus was quantified. Each individual myocyte was quantified and average cross sectional area was plotted (n = 180 myocytes from 9 field of views from 3 animals/group). # indicates *p* < 0.001 versus control. (**C**) TA CSA was displayed by fiber type distribution. (**D**) Soleus CSA was displayed by fiber type distribution. (**E**) WGA staining shows cardiac myocyte membrane staining. Images were obtained with × 10 objective (NA = 0.5). (**F**) MetSyn increased cardiac myocyte CSA and quantified data was plotted (n = 450 myocytes from 9 field of views from 3 animals/group). # indicates *p* < 0.001 versus control. Data are presented as mean ± SE.

We have previously reported hyperinsulinemia and hyperlipidemia in high-fructose-induced MetSyn animals^9^. As we expected, blood glucose levels in the high-fructose diet fed rats were significantly increased (7.37 ± 0.39 vs. 13.37 ± 0.55 mM, control vs. MetSyn, *p* < 0.05) compared with the control group rats in the normal-diet chow (Table 1). As we reported in our previous studies, we did not observe any significant increase in body mass over the 7–10 weeks diet period in MetSyn rats comparing to the control group. However, skeletal muscle mass was found to be decreased in the MetSyn group when compared to the controls, which is consistent with the common pathological phenotype in metabolic disorders^30^. Soleus and tibialis anterior (TA) muscles from MetSyn rats showed a significant decrease in muscle mass (*p* < 0.05) and in the fiber type distribution that is skewed to the smaller muscle fiber (Fig. 1A–D). In contrast, accumulation of body fat in both visceral and inguinal subcutaneous fat pad was significantly higher in MetSyn rats (Table 1). Additionally, heart weight was significantly increased in MetSyn animals and exhibited enlarged cardiac myocytes comparing with the myocytes from control rats (Table 1 and Fig. 1E, F).

**Table 1:** Effect of high fructose dietary feeding on MetSyn.

	Control (n = 18)	MetSyn (n = 18)
Body weight (g)	390.42 ± 11.43	384.39 ± 14.83
Glucose (mM)	7.37 ± 0.39	13.37 ± 0.55*
Visceral epididymal adipose fat (%)	0.75 ± 0.05	1.74 ± 0.14*
Subcutaneous inguinal adipose fat (%)	0.62 ± 0.06	1.47 ± 0.112*
Heart weight (g)	1.29 ± 0.06	1.34.6 ± 0.04*
Heart/Bwt (mg/g)	3.109 ± 0.09	3.48 ± 0.07*
Soleus (mg)	218.1 ± 8.83	170.9 ± 8.54*
Soleus/Bwt (mg/g)	0.56 ± 0.02	0.45 ± 0.038*
TA (mg)	849.9 ± 44.58	684.2 ± 57.86*
TA/Bwt (mg/g)	2.19 ± 0.07	1.78 ± 0.15*

Values are expressed as means ± SE. *p < 0.05.

### SERCA inhibition abolishes intrinsic lymphatic pumping activity

**Figure 2:**
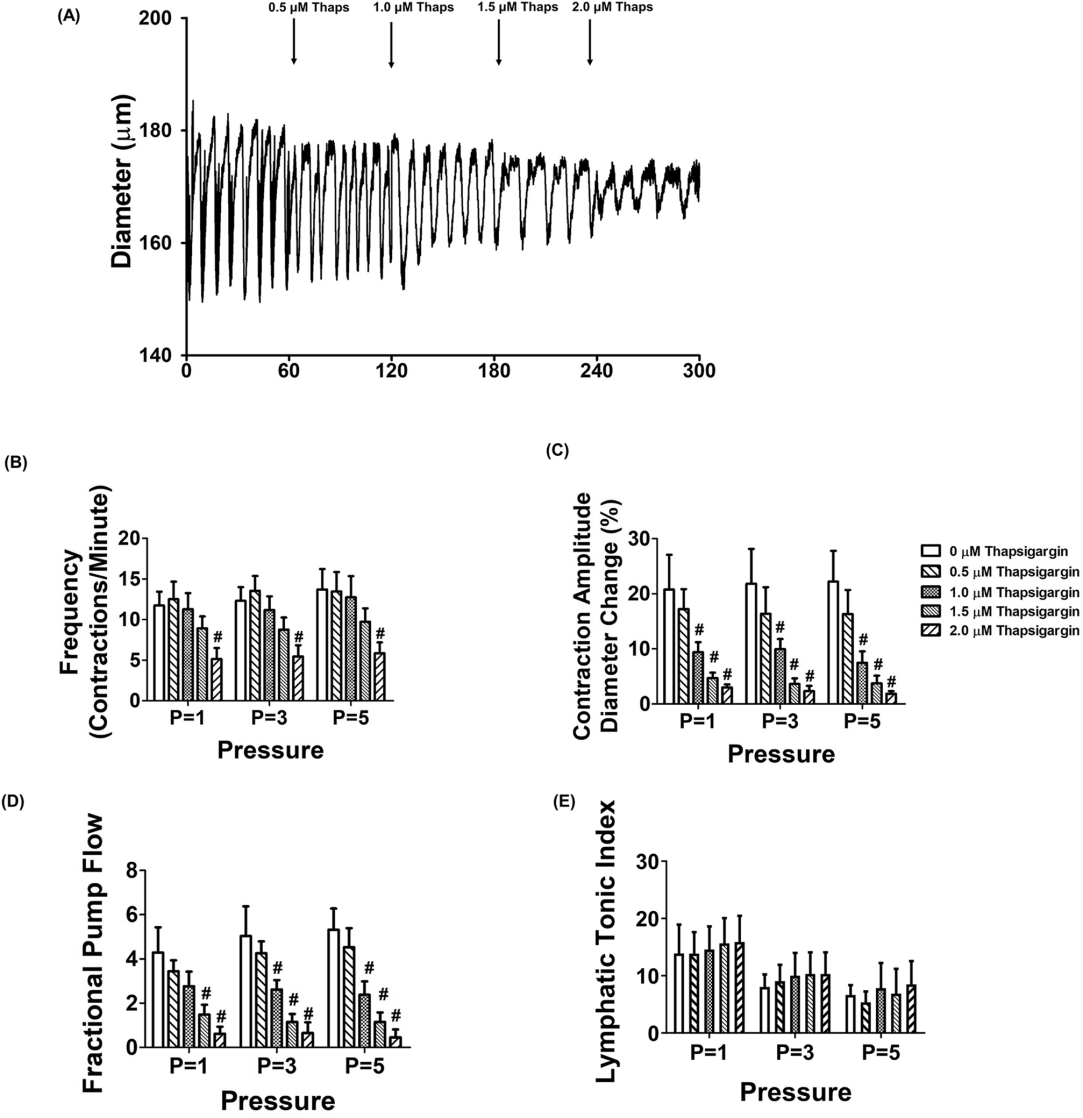
Inhibition of SERCA with thapsigargin causes impaired lymphatic pumping of rat mesenteric collecting lymphatic vessels. (**A**) An example of the lymphatic diameter over time before and after treatment with increasing concentrations of the SERCA inhibitor thapsigargin at 3 cmH_2_O. The SERCA inhibitor, thapsigargin significantly decreased (**B**) contractile frequency, (**C**) contraction amplitude, (**D**) fractional pump flow, and (**E**) lymphatic tonic index (n = 6 lymphatic vessels from 5 animals). # < 0.05 versus control (no thapsigargin). Data are presented as mean ± SE.

To assess the role of SERCA in the regulation of lymphatic vessel contractility, we examined lymphatic vessel contractions in different transmural pressures in the absence or presence of various doses of SERCA pump inhibitor, thapsigargin. Thapsigargin is an irreversible selective inhibitor of SERCA by both inhibiting its calcium binding and phosphorylation^31^. At concentrations higher than 5 μM, thapsigargin completely inhibited lymphatic vessels phasic contractions (data not shown). Therefore, we examined the effects of thapsigargin in the dose range of 500 nM to 2 μM on lymphatic vessel contractile properties. While 500 nM dose of thapsigargin did not affect the contractile frequency, amplitude, ejection fraction and fractional pump flow at any tested transmural pressures, 1.0 μM thapsigargin significantly reduced the contraction amplitude and the fractional pump flow at P = 3 or P = 5 cmH_2_O (Fig. 2C,D). Though the contractile frequency of the lymphatics was not significantly reduced when treated with 1.5 μM of thapsigargin, the pressure-dependent increases in contractile frequencies were blunted (11.73 ± 1.71, 12.3 ± 1.7, 13.7 ± 2.5 vs. 8.09 ± 1.61, 8.26 ± 1.45, 8.24 ± 1.33 contractions/min at p = 1, 3, and, 5 cmH_2_O, control vs. 1.5 μM thapsigargin respectively). Additionally, inhibition of SERCA by thapsigargin significantly reduced contractile amplitude and fractional pump flow (*p* < 0.05, Fig. 2C,D). 2 μM thapsigargin was found to severely impair the lymphatic contractions with decreased frequency and amplitude, and thus fractional pump flow (Fig. 2B–D). Thapsigargin did not affect lymphatic vessel tone at all tested transluminal pressures (Fig. 2E). Therefore, we employed 1.5 μM of thapsigargin to examine the role of SERCA activity in the regulation of lymphatic vessel contractility of MetSyn animals.

### SERCA inhibition does not reduce contractile activity in already compromised MetSyn lymphatic vessels

**Figure 3:**
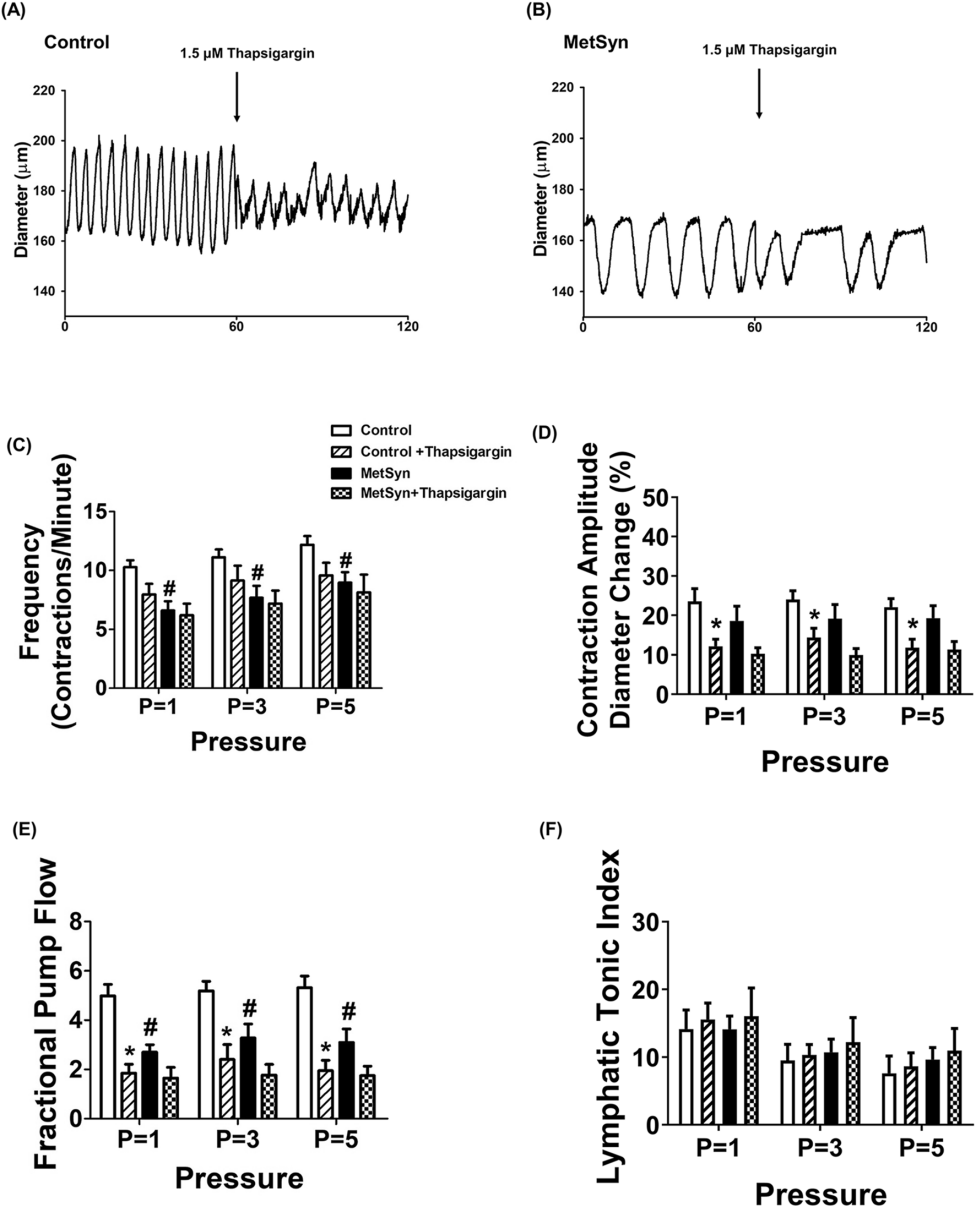
Lymphatic pump dysfunction in isolated lymphatic vessels from MetSyn animals was due to impaired SERCA activity. Representative diameter traces over 1 min period of: (**A**) control and (**B**) MetSyn lymphatic vessel at pressure 3 cmH_2_O in the presence of SERCA inhibitor. The effect of SERCA inhibitor on: (**C**) contractile frequency, (**D**) contraction amplitude, (**E**) fractional pump flow, and (**F**) lymphatic tonic index, for control (n = 12 from 6 animals) and MetSyn (n = 10 from 6 animals). # indicates *p* < 0.05 versus control groups at any value of transmural pressure. * indicates *p* < 0.05 versus each cohort controls at any value of transmural pressure. Data are presented as mean ± SE.

In control lymphatic vessels, as discussed above, the phasic contractile frequency was decreased at each employed pressure, in the presence of thapsigargin (10.27 ± 0.59 vs. 7.96 ± 0.9, 11.12 ± 0.69 vs. 9.14 ± 1.27, and 12.19 ± 0.73 vs. 9.58 ± 1.06 contractions per min, at 1, 3, and 5 cmH_2_O pressure respectively), but was not statistically significant (Fig. 3A, C, Table 2). In MetSyn animals, contractile frequency was significantly decreased compared to control animals (6.59 ± 0.8, 7.67 ± 1.02, 8.96 ± 0.89 at P = 1, 3, and 5 cmH_2_O respectively, *p* < 0.05, Fig. 3B,C, Table 2), as we have previously reported^9,10^. However, thapsigargin did not further decrease the contractile frequency of lymphatic vessels from MetSyn animals. In addition, the reduction in contractile frequency due to thapsigargin in control animals was found to be similar to the contractile frequency in MetSyn animals (Fig. 3C). Contraction amplitude was not significantly different among control and MetSyn lymphatic vessels (Fig. 3D). Thapsigargin diminished contraction amplitude significantly only in control lymphatic vessels, not in MetSyn lymphatic vessel though it showed a decrease trend (Fig. 3D). Additionally, thapsigargin significantly reduced the ejection fraction in both control and MetSyn animals at transmural pressures, P = 1, 3 and 5 cmH_2_O, while there was no significant difference between the control and MetSyn groups (Table 2). The fractional pump flow were also reduced significantly by thapsigargin in control lymphatic vessels in all transmural pressures (*p* < 0.05, Fig. 3E). While fractional pump flow were significantly decreased in the lymphatic vessels from MetSyn rats compared to the control group, thapsigargin did not further diminish these parameters in MetSyn group (Fig. 3E). Thapsigargin did not affect lymphatic tone both in control and MetSyn lymphatic vessels (Fig. 3F). A summary of all the lymphatic contractile parameters is given in Table 2.

**Table 2:** Contractile parameters for control and MetSyn mesentery lymphatic vessels presence and absence of thapsigargin (1.5 μm) or CND1163 (5 μM).

Parameters	Cohort	P = 1	P = 3	P = 5
Outer diastolic diameter (μm)	Control	136.53 ± 6.38	152.14 ± 7.1	161.58 ± 7.21
	Control Thapsigargin	133.71 ± 4.28	151.22 ± 7.01	160.5 ± 7.53
	MetSyn	127.8 ± 6.43	135.02 ± 6.92	141.95 ± 7.75
	MetSyn thapsigargin	121.37 ± 6.12	132.89 ± 6.86	141.39 ± 6.89
Contraction frequency per minute	Control	10.27 ± 0.59	11.12 ± 0.69	12.19 ± 0.73
	Control thapsigargin	7.96 ± 0.9	9.14 ± 1.27	9.58 ± 1.06
	MetSyn	**6.59 ± 0.8**^**#**^	**7.67 ± 1.02**^**#**^	**8.96 ± 0.89**^**#**^
	MetSyn thapsigargin	6.17 ± 1.09	7.16 ± 1.19	8.13 ± 1.44
Ejection fraction	Control	0.49 ± 0.04	0.48 ± 0.05	0.47 ± 0.04
	Control thapsigargin	0.26 ± 0.03*	0.27 ± 0.06*	0.19 ± 0.04*
	MetSyn	0.45 ± 0.06	0.41 ± 0.06	0.36 ± 0.04
	MetSyn thapsigargin	0.24 ± 0.04*	0.22 ± 0.03*	0.18 ± 0.03*
Fractional pump flow (min^−1^)	Control	4.98 ± 0.47	5.12 ± 0.38	5.32 ± 0.47
	Control thapsigargin	1.85 ± 0.35*	2.40 ± 0.61*	1.95 ± 0.42*
	MetSyn	**2.70 ± 0.30**^**#**^	**3.28 ± 0.56**^**#**^	**3.09 ± 0.54**^**#**^
	MetSyn thapsigargin	1.64 ± 0.43	1.77 ± 0.43	1.75 ± 0.38
Contraction amplitude (%)	Control	23.51 ± 3.28	24.01 ± 2.2	22.05 ± 2.18
	Control thapsigargin	12.20 ± 1.79*	14.40 ± 2.37*	11.85 ± 2.13*
	MetSyn	18.56 ± 3.77	19.13 ± 3.58	19.27 ± 3.16
	MetSyn thapsigargin	10.29 ± 1.51	9.93 ± 1.67	11.37 ± 2.01
Tonic index	Control	14.14 ± 2.82	9.52 ± 2.36	7.62 ± 2.55
	Control thapsigargin	15.57 ± 2.42	10.3 ± 1.57	8.63 ± 2.01
	MetSyn	14.11 ± 1.94	10.72 ± 1.94	9.64 ± 1.78
	MetSyn thapsigargin	18.03 ± 4.19	12.22 ± 3.62	10.96 ± 3.27
Outer diastolic diameter (μm)	Control	150.2 ± 17.93	164.7 ± 11.52	171.6 ± 51
	Control CDN1163	143.2 ± 17.05	162.8 ± 13.7	171.2 ± 11.06
	MetSyn	137.8 ± 11.94	146.1 ± 11.44	151.1 ± 10.54
	MetSyn CDN1163	134.2 ± 10.44	142.0 ± 9.78	148.2 ± 8.92
Contraction frequency per minute	Control	11.83 ± 1.71	12.12 ± 1.48	12.29 ± 1.33
	Control CDN1163	15.82 ± 1.8	19.83 ± 2.94*	21.68 ± 4.08*
	MetSyn	**5.32 ± 0.71**^**#**^	**6.03 ± 0.59**^**#**^	**7.08 ± 0.52**^**#**^
	MetSyn CDN1163	14.86 ± 3.41*	16.68 ± 4.02*	17.24 ± 3.87*
Ejection fraction	Control	0.38 ± 0.37	0.42 ± 0.06	0.39 ± 0.05
	Control CDN1163	0.29 ± 0.39	0.31 ± 0.07	0.28 ± 0.07
	MetSyn	0.30 ± 0.46	0.37 ± 0.06	0.32 ± 0.05
	MetSyn CDN1163	0.22 ± 0.51	0.20 ± 0.03	0.19 ± 0.03
Fractional pump flow (min^−1^)	Control	4.49 ± 0.41	4.94 ± 0.72	4.62 ± 0.98
	Control CDN1163	5.29 ± 1.34	5.49 ± 1.25	5.27 ± 1.66
	MetSyn	**2.13 ± 0.73**^**#**^	**2.16 ± 0.68**^**#**^	**2.19 ± 0.57**^**#**^
	MetSyn CDN1163	3.31 ± 0.69	3.14 ± 0.60	2.82 ± 0.64
Contraction amplitude (%)	Control	25.50 ± 4.03	29.05 ± 3.15	26.13 ± 2.82
	Control CDN1163	16.66 ± 3.65	15.8 ± 5.4	24.72 ± 6.47
	MetSyn	23.37 ± 5.9	21.66 ± 3.85	21.00 ± 4.19
	MetSyn CDN1163	16.33 ± 2.34	14.77 ± 2.28	13.71 ± 2.29
Tonic index	Control	15.38 ± 1.01	9.25 ± 0.79	7.19 ± 0.99
	Control CDN1163	19.04 ± 2.38	10.43 ± 2.03	7.29 ± 1.75
	MetSyn	14.29 ± 0.51	9.62 ± 1.27	7.83 ± 0.86
	MetSyn CDN1163	16.06 ± 2.36	11.76 ± 1.86	9.27 ± 1.56

Values are expressed as mean ± SE. * indicate significant difference between thapsigargin or CDN1163 treatment within each cohort group at any value of transmural pressure (*p* < 0.05). # indicate significant difference between control and MetSyn lymphatic vessels in control group at any value of transmural pressure (*p* < 0.05). The significant differences in values are bolded.

### Decreased SERCA2a expression in MetSyn lymphatic vessel

**Figure 4:**
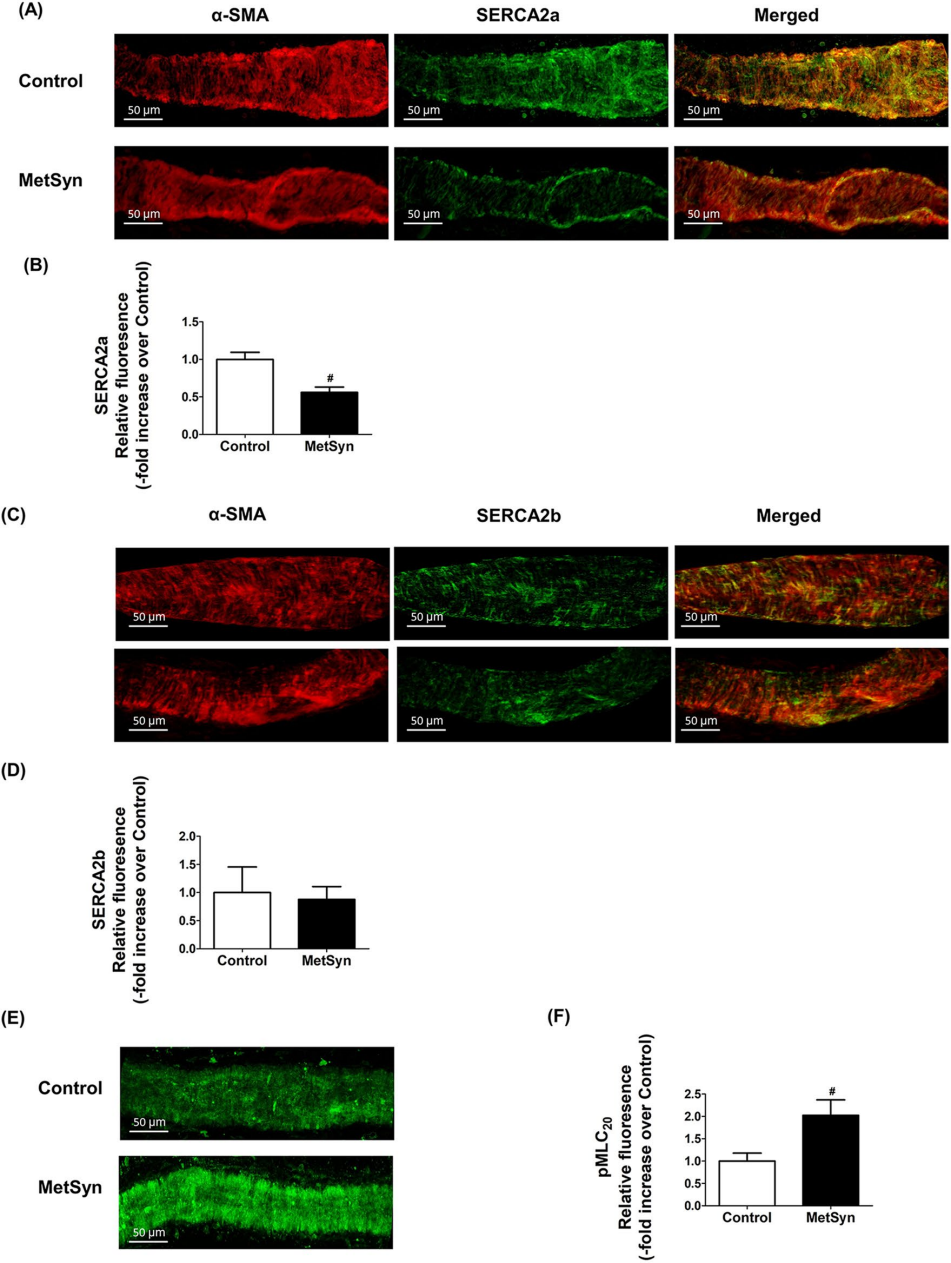
Decreased SERCA2a expression coupled with elevation of MLC_20_ phosphorylation in lymphatic vessels from MetSyn animals. (**A**) Representative images of SERCA2a and α-SMA staining for lymphatic vessels from control and MetSyn rats. Images were obtained with × 40 objective (NA = 0.9) on a confocal microscope. Scale bars, 50 μm. (**B**) Average projections were quantified and plotted for control (n = 18 fields of views from 6 vessels from 3 animals) and MetSyn (n = 30 fields of views from 6 vessels from 3 animals). Quantification of SERCA2a was plotted. # indicates *p* < 0.001 vs control. (**C**) Representative images of SERCA2b and α-SMA staining for lymphatic vessels from control and MetSyn rats. Images were obtained with × 40 objective (NA = 0.9) on a confocal microscope, and average projections were presented. Scale bars, 50 μm. (**D**) Images were quantified and plotted for control (n = 9 fields of views from 4 vessels from 2 animals) and (n = 10 fields of view from 4 vessels from 2 animals). (**E**) Representative images of MLC_20_ phosphorylation staining for lymphatic vessels from control and MetSyn rats. Images were obtained with × 40 objective (NA = 0.9) on a confocal microscope. Scale bars, 50 μm. (**F**) Average projections were quantified and plotted for control (n = 17 fields of view from 6 vessels from 3 animals) and MetSyn (n = 12 fields of view from 6 vessels from 3 animals). # *p* < 0.008 versus control. Data are presented as mean ± SE.

To assess the expression of SERCA, we performed immunofluorescence analyses on isolated mesenteric lymphatic vessels from control and MetSyn rats using striated muscle-specific SERCA2a or striated and smooth muscle isoform, SERCA2b specific antibody co-stained with α-smooth muscle actin (α-SMA) antibody. Both SERCA2a and SERCA2b positive staining were revealed on the lymphatic vessel wall. SERCA2a and SERCA2b are co-stained with α-SMA in lymphatic muscle, indicating both SERCA2a and SERCA2b are present in lymphatic muscle (Fig. 4A,C). Negative controls were performed with normal rabbit IgG (data not shown). Further quantitative analyses showed that SERCA2a expression was significantly decreased in the MetSyn lymphatic muscle compared to control group (0.56 ± 0.39 fold, *p* < 0.001, n = 9 vessels from three animals/group, Fig. 4A,B); however, there was no significant differences in the SERCA2b expression among the control and MetSyn groups. We further examined the relative levels of MLC_20_ phosphorylation in the control and MetSyn lymphatic vessels. MetSyn lymphatic vessels displayed significantly higher levels of phosphorylated MLC_20_ (*p* < 0.008, Fig. 4E,F).

### Insulin resistance impaired SERCA2 activity in LMCs

**Figure 5:**
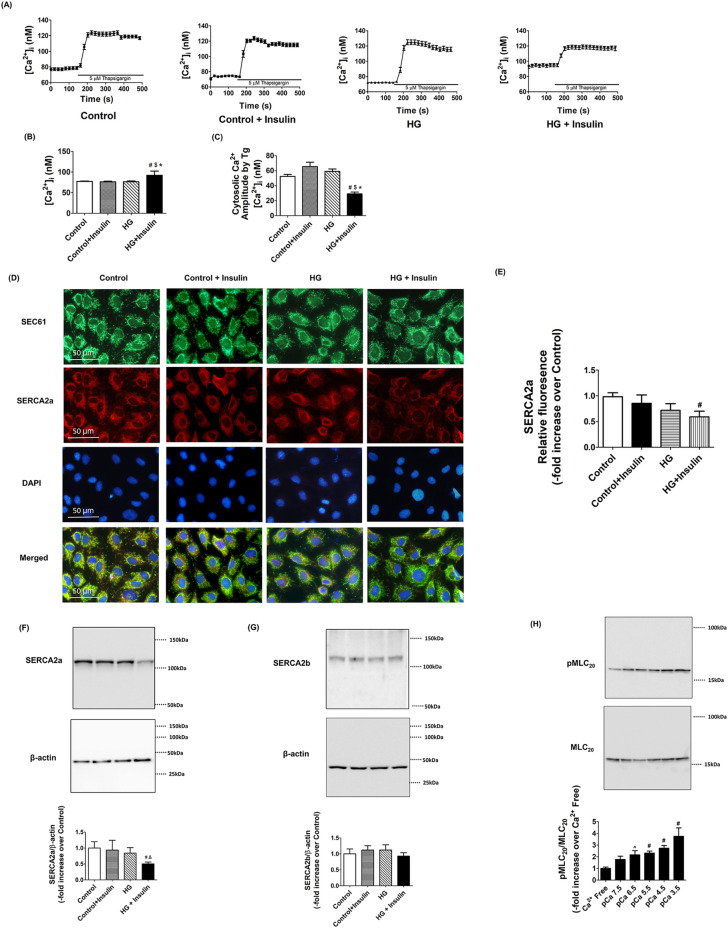
Insulin resistant conditions impaired SERCA activity and decreased SERCA2a expression in LMCs. (**A**) An example tracing shows that the SERCA inhibitor, thapsigargin increased intracellular Ca^2+^ levels in the absence or presence of high glucose and insulin (n = 100 cells from 5 different cultured LMC dishes/groups). (**B**) Basal calcium level, before thapsigargin, was quantified and plotted. # indicates *p* < 0.001 versus control. $ indicates *p* < 0.001 versus control + insulin. * indicates *p* < 0.001 versus HG. (**C**) Amplitude between peak intracellular Ca^2+^ after thapsigargin and basal was quantified for determining SERCA activity and plotted. # indicates *p* < 0.001 versus control. $ indicates *p* < 0.001 versus control + insulin. * indicates *p* < 0.001 versus HG. (**D**). Representative immunofluorescence images of lymphatic muscle cells stained with antibodies specific for the ER (SEC61, green), SERCA2a (red), and DAPI (blue). Images were obtained using × 40 objective (NA = 1.3) on a fluorescence microscope (n = 9 field of views from 3 cultured dishes/group). Scale bars, 50 μm. (**E**) SERCA2a relative fluorescence intensity was quantified and plotted. # indicates *p* < 0.032 versus control. Representative Western blots of SERCA2a (**F**) and SERCA2b (**G**) relative levels in LMCs treated with HG, insulin, or both together for 48 h. The relative expression of SERCA2a/β-actin and SERCA2b/β-actin were quantified and plotted (n = 3/group). # indicates *p* < 0.05 versus control. $ indicates *p* < 0.05 versus control + insulin. (**H**) Representative Western blots show increased free extracellular calcium concentration induced MLC_20_ phosphorylation in LMCs. The relative expression of pMLC_20_/total MLC_20_ (n = 4/group). ^ indicates *p* < 0.1 compared to Ca^2+^ free control. # indicates *p* < 0.05 versus Ca^2+^ free control. Data are presented as mean ± SE.

To address whether insulin resistance conditions in LMCs impair SERCA activity and expression, and calcium regulation, LMCs were cultured in hyperglycemia and hyperinsulinemia conditions for 48 h as described in our previous study^21^. Insulin resistant LMCs showed elevated basal intracellular Ca^2+^ levels (92.1 ± 2.64 nM) compared to controls (77.19 ± 1.01 nM, *p* < 0.001) and other groups (Fig. 5A,B). We used 5 μM of thapsigargin that prevented Ca^2+^ uptake into the endoplasmic reticulum by blocking SERCA. Thus, inhibiting SERCA contributed to significantly increase peak intracellular Ca^2+^ in LMCs. Peak intracellular Ca^2+^ and transient time to reach the peak Ca^2+^ levels were not significantly different between the groups (Fig. 5A). The amplitude between basal and peak Ca^2+^ levels in the presence of the thapsigargin was found to be significantly decreased in insulin resistant LMCs (Control, 52.36 ± 2.59 nM vs. HG + Insulin, 30.52 ± 2.42 nM, *p* < 0.001), indicating decreased SERCA activity in these cells (Fig. 5A,C). No difference was observed between groups in the peak Ca^2+^ levels when we depolarized the cells using 80 mM K^+^ (data not shown). ER specific SEC61 protein staining showed no differences in the ER morphology between the groups. SERCA2a expression was significantly lower in insulin resistant LMCs when compared to control LMCs (− 0.41 fold vs. control, *p* < 0.032) (Fig. 5D,E). Corroborating our immunofluorescence data, western blot analyses demonstrated that SERCA2a protein expression was decreased in insulin resistant LMCs (− 0.58 fold vs. control, *p* < 0.026) while SERCA2b expression remained unchanged (Fig. 5F,G). Next, we tested whether increased extracellular free calcium level in LMCs would directly influence MLC_20_ phosphorylation levels as external calcium levels affect intracellular calcium level and muscle contractility^32–34^. LMCs were grown in the media containing different free calcium concentration solution (calcium free to pCa3.5). MLC_20_ phosphorylation was found to be increased in LMCs grown under increasing extracellular Ca^2+^ concentrations and showed significant increase at pCa 6.5 or at higher pCa (*p* < 0.05, Fig. 5H).

### Activation of SERCA pump partially improves lymphatic contractile activity in MetSyn animals

**Figure 6:**
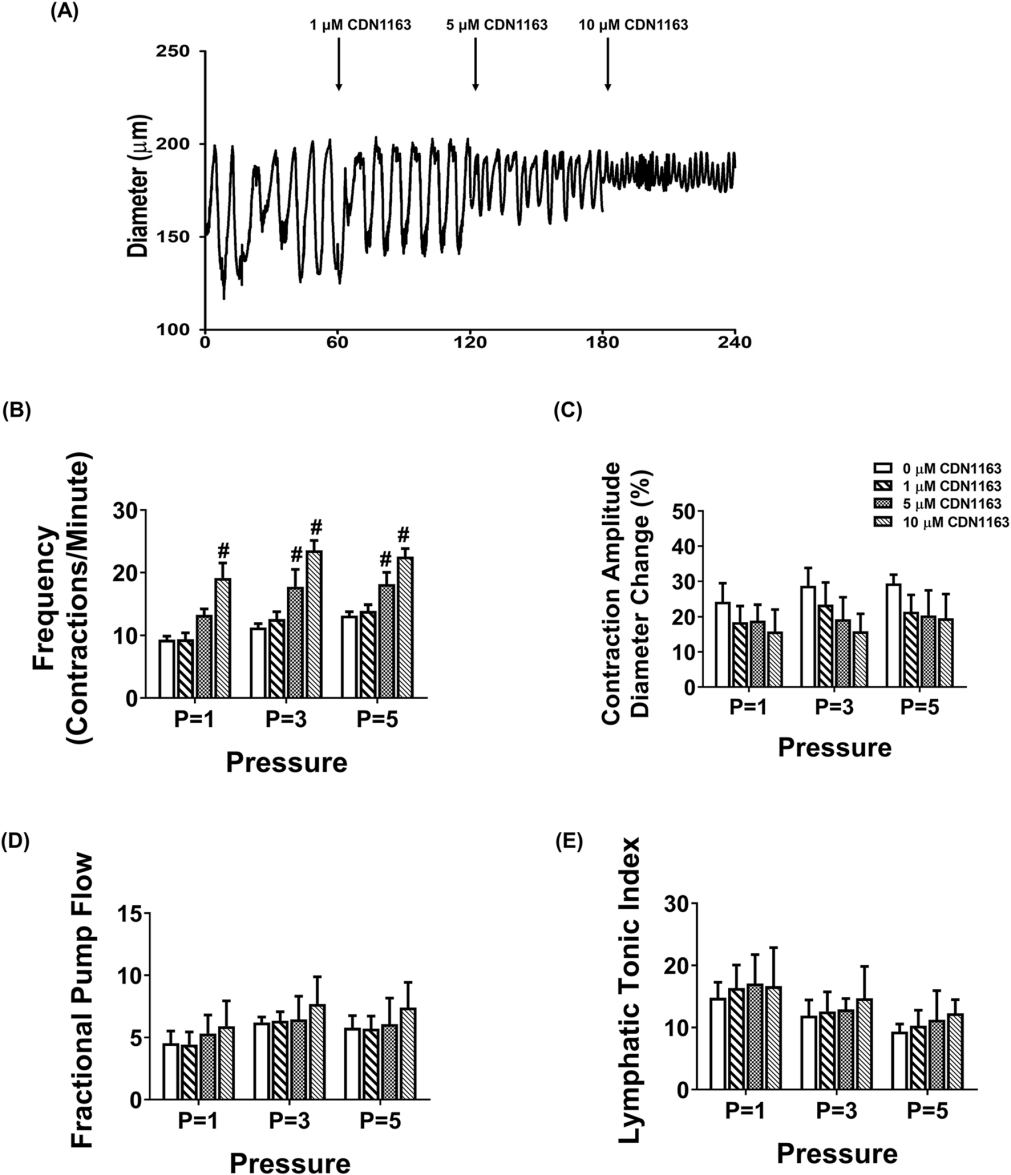

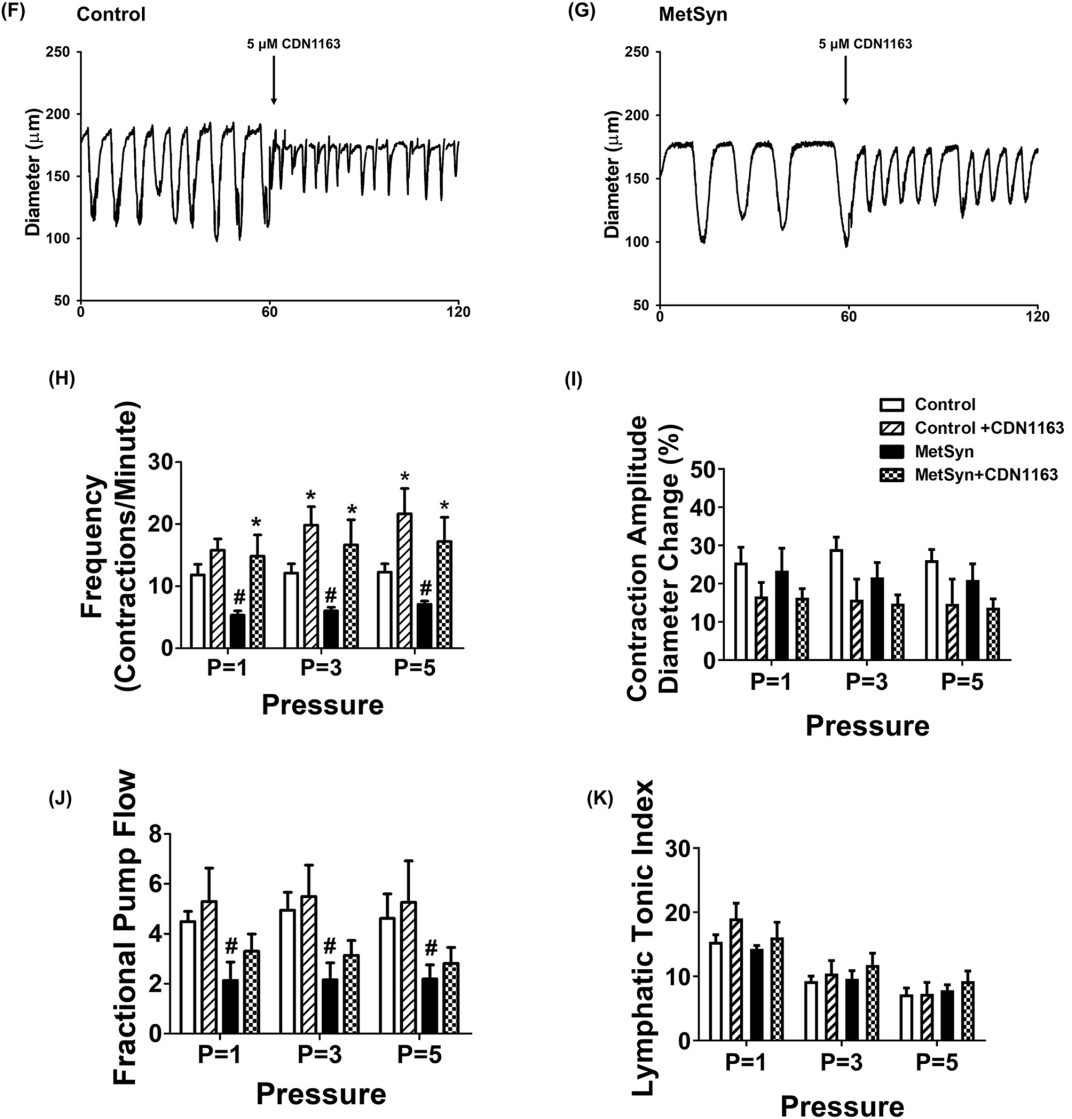
The effects of SERCA activator, CDN1163 on contraction in isolated lymphatic preparations from control and MetSyn rats. (**A**) Representative trace of control mesenteric lymphatic vessel at 3 cmH_2_O transluminal pressure with different doses of CDN1163 (n = 5 lymphatic vessels). The effects of CND1163 on: (**B**) contraction frequency, (**C**) contraction amplitude, (**D**) fractional pump flow, and (**E**) lymphatic tonic index. # indicates *p* < 0.01 versus control. Representative diameter traces of control (**F**) and MetSyn (**G**) mesenteric lymphatic vessels at pressure 3 cmH_2_O in the presence of CDN1163. The effects of CDN1163 on: (**H**) contractile frequency, (**I**) contraction amplitude, (**J**) fractional pump flow, and (**K**) lymphatic tonic index (n = 6 from 4 animal/group). # indicates *p* < 0.05 versus control groups at any value of transmural pressure. * indicates *p* < 0.05 compared to each cohort controls at any value of transmural pressure. Data are presented as mean ± SE.

CDN1163 is a small molecule that activates SERCA2 by directly binding to the SERCA2 structure and increases SERCA2 V_max_ activity^35–37^. In this study, we tested whether exogenously adding CDN1163 in the isolated lymphatic vessel preparations of MetSyn rats would improve its pumping activity. We selected different doses of CDN1163 (1, 5, and 10 μM) based on previous studies. We initially determined the effects of CDN1163 on lymphatic contractile parameters of the control vessels. While lymphatic contractile frequency was not affected in the presence of 1 μM CDN 1163, 5 μM CDN1163 significantly increased contractile frequency of lymphatic vessels at transmural pressures, 3 cmH_2_O (17.74 ± 2.7 contractions/min, *p* < 0.022) and 5cmH_2_O (18.16 ± 1.9 contractions/min, *p* < 0.016, Fig. 6B). Lymphatic contractile frequency was increased at all different transmural pressures in the presence of 10 μm CDN1163 (*p* < 0.001, Fig. 6B). Though CDN1163 did not alter significantly the contractile amplitude and tone (Fig. 6C,E), there was a trend showing a decrease in lymphatic contraction amplitude in a dose dependent manner (Fig. 6C). Ejection fraction was not significantly affected in the presence of CDN1163 (Table 2). Therefore, even if CDN1163 significantly increased contractile frequency of control lymphatic vessels, lymphatic fractional pump flow did not change at all different transmural pressures in the presence of 5 μm CDN1163 (Fig. 6D). Hence, we used 5 μm CDN1163 that showed positive chronotropic effect without negative inotropic effect (Fig. 6A–E), to test whether CDN1163 ameliorates MetSyn induced impaired lymphatic contractile activity. CDN1163 significantly improved contractile frequency of lymphatic vessels from MetSyn animals, as we found in control vessels (Fig. 6F–H). Similar to lymphatic vessels from control animals, SERCA activation did not improve fractional pump flow significantly in the lymphatic vessels from MetSyn group (Fig. 6J). Though there was a tendency of reduction in contraction amplitude by CDN1163 on both control and MetSyn lymphatic vessels, the differences were not significant (Fig. 6I). Additionally, we do not see any noticeable difference in lymphatic tone by CDN 1163 in both control and MetSyn lymphatic vessels (Fig. 6K). The other calculated lymphatic contractile parameters of control and MetSyn rats are detailed in Table 2.

## Discussion

**Figure 7:**
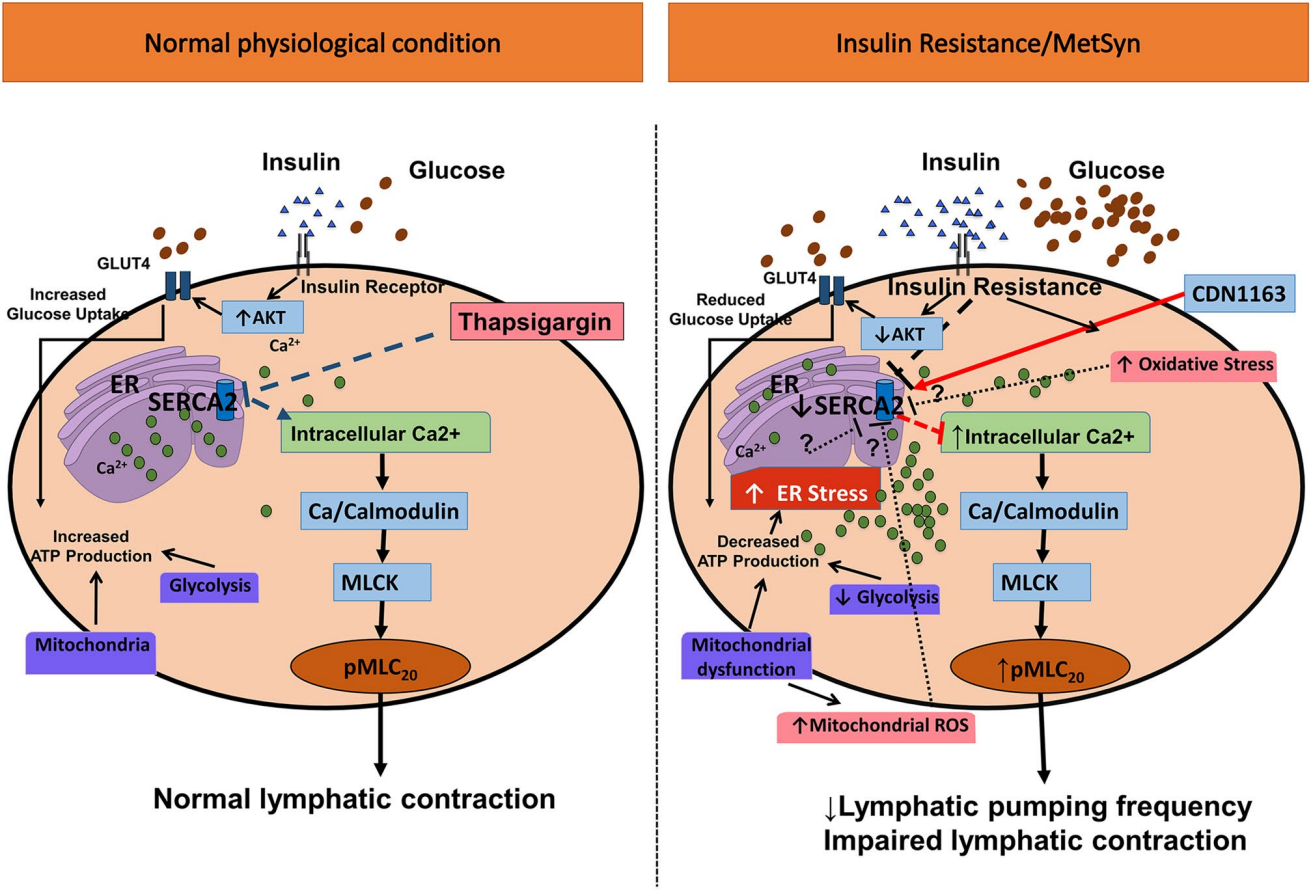
Schematic representation of lymphatic vessel contraction under normal and MetSyn/insulin resistant conditions. In normal physiological condition, Ca^2+^ homeostasis is critical for lymphatic pump regulation. The inhibition of SERCA directly diminishes lymphatic contractile activity and it is resulted in increasing intracellular Ca^2+^. Elevated intercellular Ca^2+^ induce MLC_20_ phosphorylation, a key contractile regulatory molecule that also influences lymphatic contractions. Insulin resistance in LMCs or high fructose diet-induced MetSyn condition in rats decreases SERCA2a expression and SERCA activity, disturbing intracellular Ca^2+^ homeostasis and impairs lymphatic phasic contraction. Elevated intracellular Ca^2+^ promotes MLC_20_ phosphorylation via Ca^2+^/calmodulin and MLCK pathway^82^ in insulin resistant LMCs. Normal insulin signaling in LMCs mediates its bioenergetics via proper glucose uptake and glycolysis and mitochondrial function^21^. Disrupted insulin signaling in insulin-resistant LMCs inhibits glucose uptake, induces mitochondrial dysfunction, and decreases bioenergetics^21^. Imbalanced cellular bioenergetics status causes energy stress or endoplasmic reticulum stress that may decrease SERCA expression or activity in insulin resistant LMCs and in MetSyn animals, which is not yet tested.

The data presented in this study provide the first evidence that the striated muscle-specific, SERCA2a pump that is present in lymphatic muscle modulates the lymphatic pumping activity by regulating phasic contractile amplitude and frequency, but not the lymphatic tone. Additionally, diminished lymphatic contractile activity in the vessels from the MetSyn animal is associated with the decreased SERCA2a expression and SERCA2 dysfunction. SERCA activator, CDN1163 significantly improved the contractile frequency and partially restored lymphatic pump function in MetSyn mesenteric lymphatic vessels. Additionally, our data demonstrate that reduced SERCA2a expression resulted in impaired Ca^2+^ homeostasis in insulin resistant LMCs. Collectively, these data suggest MetSyn conditions diminished SERCA2a expression and SERCA activity in lymphatic muscle, consequently reduced lymphatic contractile activity in MetSyn rats (Fig. 7).

In keeping with previous studies, we found that the MetSyn animals had increased glucose levels and other characteristics of MetSyn conditions, such as elevated levels of triglycerides and cholesterol^9,38,39^. Further, we observed increased subcutaneous and visceral body fat with decreased muscle mass (Table 1 and Fig. 1). Cross sectional analysis of the TA and soleus muscle from MetSyn animals showed left skewed in fiber type size distribution graph, indicating muscle atrophy (Fig. 1C,D). The average cross-sectional area was significantly smaller in both MetSyn TA and soleus muscle (Fig. 1A–D). The muscle atrophy in metabolic diseases is defined by the pathological term ‘sarcopenic obesity’^40–42^, and altered body composition signifies the important pathological aspects of our MetSyn model. In addition, the muscle loss in obesity or metabolic disease could result from chronic inflammation^43–45^. Our previous data showed increased proinflammatory signaling in MetSyn mesentery bed with activation of M1 macrophages^10^. In addition, dietary endotoxin, LPS, that also cause insulin resistance, altered inflammatory immune response in the lymphatic mesenteric bed^46^. Hence, we speculate that muscle atrophy in fructose diet-induced MetSyn rats could be due to systemic inflammatory effects. Cardiac hypertrophic remodeling is one of the prevailing pathological features of metabolic disorders due to chronic systemic inflammation^47–49^. Our data showed that hearts from fructose-induced MetSyn rats displayed increased heart weight and enlarged cardiomyocyte cross- sectional area (Table 1 and Fig. 1).

SERCA is one of the key regulators of striated and smooth muscles’ contractions by regulating intracellular Ca^2+^ levels^50,51^. While the role of SERCA in cardiac, skeletal, and smooth muscle have been largely investigated, very little is known about the role of SERCA in lymphatic muscle. One study showed that a SERCA inhibitor, cyclopiazonic acid (7 μm) completely blocked lymphatic function in bovine mesentery lymph vessels at all different transmural pressures^25^. However, whether the inhibitory effects of SERCA was due to chronotropic or inotropic effects had not been tested. We employed thapsigargin that exhibits increased inhibitory effects on SERCA compared to other inhibitors^31^. Our data showed that inhibition of SERCA significantly reduced lymphatic contractile frequency, amplitude and thus, diminished fractional pump flow and ejection fraction (Fig. 2 and Table 2). These results imply that SERCA is an important mediator for lymphatic pumping by regulating phasic contractile activity, not by regulating lymphatic tonic contraction.

Diminished SERCA activity in metabolic diseases has been well established in various tissues, including cardiac, skeletal and smooth muscles, and in liver tissue. Consistent with our previous findings^9,10^, lymphatic contractility indexes, frequency of contraction and lymphatic fractional pump flow, were significantly reduced in MetSyn rats when compared to control group (Fig. 3C,E). Our data showing that the SERCA inhibitor, thapsigargin significantly reduces the frequency and amplitude of contractions of lymphatic vessels from control group, but not the vessels from MetSyn animals (Fig. 3C,D), suggesting that SERCA activity is already diminished in MetSyn lymphatic vessels. Ejection fraction was significantly lowered in the presence of thapsigargin compared to each cohort groups (Table 2) and this might be due to the similar level of contractile amplitude between control and MetSyn.

Three distinct genes encoding SERCA 1, 2, and 3 produce more than 10 isoforms that are expressed in various muscles and non-muscle cells^52^. SERCA1a and b are predominant isoforms in skeletal muscle and SERCA3s are expressed in various non-muscle tissues. SERCA2a is predominantly expressed in both skeletal muscle type 1 fiber and in adult cardiac muscle; while, SERCA2b is considered one of the major isoforms in smooth muscle cells^52,53^. Here, we report that lymphatic muscle cells express both SERCA2a and SERCA2b isoforms (Figs. 4A–D, 5D–G), further supporting our previous finding that lymphatic muscle presents a unique combination of muscle cell types that express both cardiac and smooth muscle contractile and regulatory proteins^12,17–19^.

Previous studies in different metabolic disease models have shown a decreased SERCA activity in cardiac and vascular tissues, yet there were inconsistent results whether diminished SERCA activity resulted from decreased levels of SERCA expression or were independent of SERCA levels. Decreased SERCA2a protein expression was found in cardiac muscle coupled with impaired cardiac contractility in diabetic cardiomyopathy and in db/db mice^28,29,54^. Additionally, SERCA2a protein levels decreased in vascular smooth muscle cells from Type 1 and Type 2 diabetes animal models^27,55^. In contrast, studies have reported that there were conformational changes, altered SERCA regulatory molecules, or diminished SERCA activity without decreased SERCA expression in the cardiac tissues from db/db mice and in an insulin resistant rat model^56,57^. We found that SERCA2a expression was diminished significantly in MetSyn lymphatics whereas SERCA2b expression was similar between control and MetSyn groups (Fig. 4A–D). Thus, the decrease response to thapsigargin in MetSyn lymphatics that we observed could be due to the decrease levels of SERCA2a in lymphatic vessel.

One critical question we have not addressed is that how loss of expression/activity of SERCA result in a decreased lymphatic contractile frequency in MetSyn animal. It is well established in cardiac and smooth muscle literatures that intracellular calcium homeostasis that is primarily regulated by SERCA, ryanodine receptor, L-type Ca channel, Na^+^/Ca^2+^ exchanger, and T-type calcium channel, plays an imperative role in regulating cardiac and smooth muscle contractile dynamics^58,59^. However, only limited studies on mechanisms of calcium regulation are available in lymphatic muscle. Lee & von der Weid have shown that T type Ca^2+^ channel mediates chronotropy; whereas L-type Ca^2+^ channel controls inotropic contraction of lymphatic vessel^24^. Additionally, while L-type calcium channel is partially responsible for differential contractile activity of mouse lymphatic vessels from various regions of the body, it controls both frequency and strength of phasic contractions in lymphatic vessel^15,60^. In the presence of ryanodine that inhibits ryanodine receptor, lymphatic vessels showed a decrease in contractile frequency and amplitude, without a change in tonic contraction^61^, suggesting critical roles of endoplasmic reticulum stored calcium in phasic contraction. Our data show that inhibition of SERCA by thapsigargin in lymphatic vessels diminished both phasic contractile frequency and amplitude without affecting the tone. Additionally, a decrease in SERCA expression and/or activity in MetSyn lymphatics resulted in diminished phasic contraction frequency and a tendency for reduced contraction amplitude with no change in lymphatic tone. Primary role of SERCA pump is reuptake of intracellular calcium back to sarcoplasmic reticulum, which is essential for muscle relaxation. Notably, lymphatic vessel relaxation is critical for allowing filling the lymph, consequently for efficient lymphatic pumping activity^62^. Hence, a decrease in SERCA expression/activity either by thapsigargin or by MetSyn condition diminished lymphatic muscle phasic contraction. In addition, it also resulted in an increase in intracellular calcium, which induced Ca^2+^-dependent myosin light chain_20_ phosphorylation^17,18^. Increase in intracellular calcium levels also regulate voltage-gated sodium and potassium channels^63^. Taken together, we propose that increase in intracellular calcium in lymphatic muscle could disrupt the homeostasis of other ions, such as K^+^, Cl^−^ or Na^+^, by modulating respective ion channels in lymphatic muscle and consequently, it would change membrane potential and contractile frequency. Further studies are warranted how disruption in calcium homeostasis in lymphatic muscle influences other ion channels and thereby modulates phasic contractile frequency.

Decrease SERCA pump activity in lymphatic muscle would increase the cytosolic Ca^2+^ in LMCs. Our cell culture data support this notion; results showed that in the basal condition, intracellular Ca^2+^ levels were elevated in insulin resistant LMCs. Additionally, amplitude between peak and basal calcium levels was significantly decreased in insulin resistant LMCs, indicating decreased SERCA activity in LMCs treated with glucose and insulin. Increase in intracellular Ca^2+^ will promote MLC_20_ phosphorylation via Ca^2+^/calmodulin-MLCK pathway^22^. Increased phosphorylation of MLC_20_ levels we observed in lymphatic vessels from MetSyn rats (Fig. 4E,F) corroborate with our previous finding that insulin resistant condition induced MLC_20_ phosphorylation in LMCs^21^. Thus, we propose that increased intracellular Ca^2+^ due to decreased SERCA2 expression and activity in LMCs of MetSyn animals lead to an increase in MLC_20_ phosphorylation that causes a reduction in the diameter of lymphatic vessels and consequently, poor lymphatic function in MetSyn conditions that we previously reported^9,10^. Since Rho kinase regulates myosin light chain phosphatase (MLCP), which is one of the critical regulators of MLC_20_ phosphorylation^64,65^ and Rho kinase activity is increased in MetSyn condition^66,67^, we examined the phosphorylation levels of MYPT1, a subunit of MLCP, in insulin resistant LMCs. Results showed the relative levels of MYPT1 phosphorylation were not significantly changed in insulin resistant LMCs (Data not shown). We acknowledge the fact that these data are from LMC culture model and the regulation of MLC_20_ phosphorylation in MetSyn lymphatic tissue could be different from the cell culture model. It has previously shown that Rho kinase ROCK regulates both tonic and phasic contractions of lymphatic vessels^64^. Therefore, further studies are warranted to study the roles of Rho kinase and MLCP in the regulation of MLC_20_ phosphorylation and lymphatic contractility in MetSyn animals. In addition, isoforms of SERCA in lymphatic endothelial cells and its distinct roles in the modulation of flow-mediated Ca^2+^ release in MetSyn needs to be investigated^34^.

Increasing SERCA expression or activity has been shown to provide protective effects on cardiac muscle and vascular smooth muscle cells in several metabolic diseases^26,29,54,55^. A small molecule, CDN1163, activates SERCA by allosteric mechanism and improves Ca^2+^ homeostasis both in vivo and in vitro^35–37^. Our data show that SERCA activation significantly improves the lymphatic contractile frequency in MetSyn lymphatic vessels similar to control lymphatic vessels (Fig. 6F–H). Though the fractional pump flow was not significantly improved by SERCA activator in MetSyn lymphatic vessels, fractional pump flow was not significantly lower than control lymphatic vessels, suggesting partial improvement of contractile activity of MetSyn lymphatic vessels by SERCA activator. This partial improvement by SERCA activation in MetSyn group might be due to reduced SERCA2a expression in MetSyn lymphatic muscle.

In summary, the current study demonstrates that SERCA2 activation plays an important role in modulating lymphatic contractile function by regulating chronotropic and inotropic effects. The rat mesenteric lymphatic muscle expresses both SERCA2a and SERCA2b isoforms. MetSyn conditions decreased the levels of SERCA2a expression and impaired Ca^2+^ regulation in LMCs that are coupled with increased MLC_20_ phosphorylation. The impaired lymphatic pumping activity in MetSyn is due to diminished SERCA activity and activating SERCA partially improves contractile activity of lymphatic vessels from MetSyn rats. Therefore, it is possible that SERCA2 agonist could be used as a therapeutic strategy in enhancing lymphatic function in MetSyn or other metabolic diseases.

## Materials and methods

### Materials

Phospho MLC_20_ (Ser19), total MLC_20_, β-actin antibodies were purchased from Cell Signaling Technology (Danvers, MA, USA). SERCA2a and SERCA2b antibodies were purchased from Badrilla Ltd (Leads, UK). Fura-2am, Fura-2am calibration kit, Alexa Fluor secondary antibodies, DMEM/F12, DMEM, fetal bovine serum (FBS), triple antibiotics (penicillin, streptomycin, and Amphotericin B), Prolong Gold antifade mounting medium with DAPI, WGA dye, and Alexa Flour 488 antibody labeling kit were purchased from Thermo Fischer Scientific (Waltham, MA, USA). SEC61A antibody was purchased from Abcam (Cambridge, MA, USA). DMSO, thapsigargin, CDN1163, bovine serum albumin, and α-SMA antibody were purchased Sigma Aldrich (St Louis, MO, USA). All other chemicals and reagents were from Sigma Aldrich (St. Louis, MO, USA), otherwise we indicated specifically.

### Animal handling

Fifty-two male Sprague–Dawley rat weighing 150–180 g were ordered from Charles River for induction of the MetSyn. Twenty rats were given a high fructose diet (60% fructose, ID89247 Harlan Teklad, Envigo, Indianapolis, IN, USA), for 7–10 weeks to induce the MetSyn, while the remaining rats were given standard rodent chow. Water and each respective feed were available ad libitum. Rats from the control group were utilized for testing the effects of different dosage of thapsigargin, DMSO, and CDN1163, a SERCA activator (n = 12). Rats from the control and MetSyn group were used for isobaric functional analysis of mesenteric lymphatic vessel contractility (n = 14/group). The remaining 6 rats were used for IHC and RNA analysis. All animals were housed in a facility with 12-h light–dark diurnal cycle, accredited by the Association for the Assessment and Accreditation of Laboratory Animal Care and maintained in accordance with the policies defined by the Public Health Service Policy for the Humane Care and Use of Laboratory Animals. All protocols had been approved by the University Laboratory Animal Compliance Committee at Texas A&M University prior to the commencement of the study.

### Determination of metabolic parameters

High fructose diet generates a rat model of MetSyn as characterized by high insulin, glucose, triglycerides, and glucose levels along with impaired insulin sensitivity^38,68–70^. We also confirmed the development of MetSyn conditions by measuring plasma insulin, triglyceride, inflammatory cytokines in mesentery and cecum histopathological changes^9,10^. To confirm the pathology of high fructose diet-induced MetSyn we measured blood glucose using a glucometer and test strips before surgery. Heart was isolated, weighted, and then the left ventricle was dissected for measuring cardiac hypertrophy. Quadriceps, tibial anterior (TA), and soleus muscles were isolated and weighed for assessing sarcopenic obesity, which is systemic pathology due to metabolic disorders. Inguinal subcutaneous adipose tissue (SWAT) and epididymal white adipose tissue (EWAT) were dissected to determine the percent of subcutaneous and visceral fat.

### Lymphatic vessel isolation and functional analysis

Rat mesenteric collecting lymphatic vessel isolation and cannulation were performed to test isobaric lymphatic pumping activity as described in our previous studies^9,10,18^. Rats were anesthetized with Innovar-Vet (0.3 ml/kg), which is a combination of a Droperidol-fentanyl solution (Droperidol 20 mg/ml, fentanyl 0.4 mg/ml), and diazepam (2.5 mg/kg) intramuscularly. A midline excision was made and a loop of ileum was carefully exteriorized. Lymphatic vessels were carefully dissociated from the surrounding adipose tissue to prevent excess bleeding and use the following three criteria for defining lymphatic vessels: (a) no blood filling in the lumen and colorless vessels, (b) smaller diameters compared to other corresponding blood vessels, approximately 80–150 μm with thin lymphatic muscle layer, and (c) exhibiting spontaneous contractile activity. Vessels were maintained in DMEM/F12 media at 37 °C. Mesenteric lymphatic vessels were cannulated onto matched pipettes in a CH-1 chamber (Living Systems Instrumentation, St. Albans, VT, USA) and attached to separate pressure reservoirs. Vessels were incubated in DMEM/F12 or DMSO vehicle contained DMEM/F12 until it reached temperature and equilibrated at pressure 3 cmH_2_O for 30 min. After the equilibration, the vessel was recorded for 5 min at 1 cm, 3 cm, and 5 cm H_2_O of pressure. Vessels were incubated in the presence of DMSO (10–30 mM), thapsigargin (1.0–2.0 μm), or CDN1163 (1–10 μm). At the end of each experiment, the bath solution was replaced with calcium-free albumin physiological saline solution (APSS) and maximal diameter at each pressure recorded. Lymphatic outer diameters were traced for each 5 min video capture with vessel wall-tracking software developed and provided by Dr. Michael J. Davis at the University of Missouri-Columbia^71^. Briefly, outer lymphatic vessel diameters were tracked 30 times per second, providing a tracing of diameter changes throughout the periods of systole and diastole. End diastolic diameter (EDD), end systolic diameter (ESD), and contraction frequency values were directly obtained from the tracings. From the lymphatic diameter tracings, the following parameters that are analogous to those used when evaluating cardiac pump function have been calculated as previously described^9,15,72,73^.

Phasic contraction amplitude = (EDD − ESD/D_MAX_) × 100; Ejection Fraction (EF) = (EDD^2 ^ − ESD^2^/EDD^2^); Lymphatic Tonic Index = (D_MAX_ − EDD/D_MAX_) × 100; Fractional Pump Flow (FPF) = EF × contractile Frequency (an index of lymph pump flow). D_MAX_ represents the maximum passive diameter (obtained after incubation with calcium-free APSS solution) at a given level of intraluminal pressure at the end of the experiment.

### LMC culture and treatments

Primary rat mesenteric LMCs were obtained from mesenteric tissue explants of male Sprague–Dawley rats, as we have described in previous publications^12,21^. Briefly, 2–4 mesenteric lymphatic vessels were isolated under aseptic conditions from Sprague Dawley rats within a laminar hood. The vessels were cut into small segments (2–3 mm) and each small segment was transferred to a 35 mm tissue culture dish containing high-glucose Dulbecco’s modified Eagle’s medium (DMEM) supplemented with 10% FBS and 1% antibiotic/antimycotic solution (penicillin/streptomycin). The vessel segment was attached by gently pressing the two ends of the vessel onto the plastic dish surface with forceps. The culture dish containing the vessel was then carefully placed into an incubator maintained at 37 °C and 10% CO_2_. Lymphatic muscle cells, LMCs (~ 50 cells) migrated out from the vessel in 3–7 days, after which the lymphatic vessel-segment was removed from the petridish in the aseptic condition. At this condition, lymphatic endothall cells are not able to migrate and survive, whereas LMCs will sprout out from the vessel.

We then allow the sprouted LMCs further to proliferate and once the plate becomes confluent, the cells were then trypsinized and designated as passage 1 (p1) and either split into more plates and maintained in the incubator or frozen and cryo-preserved. We have previously shown that LMCs comprise of a unique combination of cardiac and smooth muscle components^12^. We routinely analyze each batch of LMCs at stages p2–p6 for expression of both cardiac and smooth muscle phenotype, using cardiac troponin C, cardiac β myosin heavy chain, alpha smooth muscle actin, SM22, and caldesmon antibodies. Phenotypes of LMCs were also verified using qRT-PCR with α/β-myosin heavy chain, cardiac troponin, and α-smooth muscle actin as we have previously described^12^. Cells were not used beyond passage 6. LMCs were cultured in DMEM, containing 10% FBS, and 1% triple antibiotics and maintained at 37 °C in 10% CO_2_ incubator. LMCs were plated in 24-well culture plates and then grown to ~ 70–80% confluence. The cells were serum starved for 24 h and treated with 5 mM glucose (control) or high glucose (HG; 25 mM), with or without insulin (100 nM) for 48 h as we reported in a previous study^21^. To test the effects of free intracellular Ca^2+^, different concentrations of pCa solutions (between pCa 7.5 and 3.5) were prepared based on Ca free DMEM using a software, MaxChelator as described^74,75^. After serum starvation, LMCs were treated with Ca^2+^ free DMEM and different concentrations of free calcium (pCa 7.5–3.5) for 30 min. Proteins were isolated as described in our previous studies^21,46^.

### Intracellular Ca^2+^ measurement in LMCs

Intracellular Ca^2+^ levels were measured using fura-2AM in phenol-free DMEM media as described^76–78^. LMCs were plated in glass bottom chamber and treated when LMCs reached 60% confluence. Cells were loaded with Fura-2AM (2 μM) in the dark for 30 min at 37 °C. Cells were then washed with phenol-free DMEM and incubated for another 30 min for de-esterification. Pairs of fluorescent images were taken by exposure to 340- and 380 nm double excitation with interference filters at selected wave length (Lamda DG-5, Sutter Instruments, Novato, CA, USA) and 510 nm emission wavelengths using an epifluorescence microscopy system (Nikon Eclipse Ti, Nikon, Melville, NY USA). The individual traces of fura-2AM dye from multiple LMC region of interest, ROI were simultaneously measured from a field of view (~ 20 ROIs/plate). The fura-2 fluorescence ratio was collected for each cell throughout the experiments (NIS-Elements software, Nikon, Melville, NY, USA). Background fluorescence was determined before the start of the experiment. Background fluorescence was subtracted and fluorescence ratios of images at 340 and 380 nm were determined. Calcium levels were measured using fura-2AM calcium imaging calibration kit (Thermo Fisher) where it had zero to 10.0 mM CaEGTA (1.35 μM free calcium buffer) prior and post experiments. The interrelationship of the free Ca^2+^ concentration and the ratio was calculated according to manufacturer’s guideline^79^. Basal Ca^2+^ levels were measured in phenol free-DMEM. Voltage-gated calcium channel were activated by depolarization with high (80 mM) K^+^ solution. SERCA activity was assessed with thapsigargin (5 μm).

### Immunofluorescence analyses

To determine the relative levels of SERCA2a, SERCA2b, and MLC_20_ phosphorylation, rat mesenteric lymphatic vessels were prepared for immunofluorescence as indicated in previous studies^18,46^. Multiple adjacent lymphatic vessels were isolated and fixed in 2% paraformaldehyde for 60 min at room temperature. After fixation, vessels were washed in PBS and permeabilized for 5 min in − 20 °C methanol. Vessels were then blocked in 5% goat serum (with 1% BSA) for 1 h, subsequently cut in half with one part being used for the experimental treatment and the other part serving as the negative control. Vessels were incubated with SERCA2a (A010-23, Badrilla, Leads, UK), SERCA2b (A010-24, Badrilla, Leads, UK), α-Smooth muscle actin (A2547, Sigma-Aldrich, St. Louis, MO, US), or phospho MLC_20_ (3,645, Cell signaling, Beverly MA, US) antibody in blocking solution overnight. Vessels were then washed and incubated with host matched secondary antibody (1:200) conjugated with Alexa Fluor 488 or Alexa Fluor 594 after washed three times. Vessels were then washed three times and mounted using ProLong gold antifade mounting media. Vessels were scanned in 0.5-μm z-axis steps using PLAPLSM 40 × objective (NA = 0.9), Olympus IX-71 inverted microscope with Fluoview 300 confocal scanning. Average projections of series sections were reconstructed and quantified using Image J software^80^. Negative control vessel segments were subjected to the same procedures as the experimental treatments except that negative controls were incubated with corresponding host IgG instead of the primary antibody. Negative controls were scanned at the same microscope settings as the experimental treatments to allow for valid comparison of the relative fluorescent intensities.

### WGA staining

Membrane specific dye, wheat germ agglutinin (WGA), was used to test MetSyn induced myopathies as described elsewhere^81^. Frozen OCT block of TA, soleus, and left ventricles were cut 10 micron thickness using cooled cryostat (Leica CM1850, Leica Biosystems Inc., Buffalo Grove, IL, USA) at − 20 °C. Sections were fixed in cooled acetone (− 20 °C) for an hour. The slides were then washed three times in PBS for 5 min and incubated with WGA for 10 min at room temperature. Sections were washed and mounted using ProLong gold antifade mounting media. Images were taken with Olympus BX41 fluorescence microscope using UPlanApo × 10 (NA = 0.4) or UplanApo X20 objective (NA = 0.7) (Olympus America, Melville, NY, USA) at an excitation peak of 545 nm with an emission spectral peak of 610 nM.

### ER and SERCA2a staining in LMCs

Immunofluorescence experiments in LMCs were performed as described earlier, with some modification^21^, to determine SERCA2a expression and localization on ER. Briefly, the treated LMCs were fixed with 2% paraformaldehyde and permeabilized with cooled methanol for 4 min at 4 °C. Cells were then blocked with 1% BSA with 5% goat serum for 1 h at room temperature. Cells incubated SERCA2a antibody (1:100) in blocking buffer for two hours at room temperature. After three washes, cells were incubated with Alexa Fluor 594 (goat anti-rabbit, 1:200) for 1 h at room temperature in the dark. Since SEC61, specific to ER protein antibody is also from the rabbit, we used commercially pre-labeled SEC61 antibody (ab205794, Abcam, Cambridge, MA, USA), generated with an antibody labeling kit (A20181, Thermo Fisher, Waltham, MA, USA) and applied after SERCA2a antibody. Cell were counterstained for the nucleus and mounted using Prolong Gold antifade mounting medium with DAPI. Images were taken with Olympus BX41 fluorescence microscope using UPlanFLN X40 objective (NA = 1.3) (Olympus America, Melville, NY, USA) and SERCA2a fluorescence intensity was measured with Image J.

### Protein isolation and Western blot analysis

Protein expression was quantified by Western blot analysis^21,46^. In brief, LMC protein lysates were prepared from LMCs and separated onto a 4–20% gradient SDS–polyacrylamide gel and transferred, and Western blot analysis was performed. SERCA2a, SERCA2b, MLC_20_ phosphorylation, MLC_20_, and β-actin (1:1,000) were used. Membranes were incubated with the appropriate horseradish peroxidase-conjugated secondary antibodies. Protein detection was conducted with an enhanced chemiluminescence system and visualized via image processor Fuji LAS-4000 Mini (GE Healthcare Bio-science, Pittsburg, PA, USA). β-actin expression was used as the loading control. Densitometric analyses was carried out using Image J (National Institute of Health, NIH, Bethesda, MD, USA). For quantification, experiments were repeated three times for each sample, and the resulting means ± SE were calculated (n = 3/group).

### Statistical analysis

Animal characteristics (e.g., bodyweight, glucose level, % fat, muscle weight, cross sectional area, etc.,) were analyzed by independent Student’s *t *tests after performing Leven’s test for equal variance. Different dose effects of thapsigargin and CDN1163 in lymphatic contractility parameters were analyzed using One-way ANOVA with Dunnett’s post hoc test to find the treatment dose differences compared to control. Control and MetSyn lymphatic contractile parameters in the presence or absence of thapsigargin or CDN1163 were analyzed using Two-way ANOVA with Bonferroni’s post hoc test to examine the interaction and main effects between two factors: diet and treatment (i.e., thapsigargin,CDN1163) . When we found a main effect or interaction, we performed follow-up ANOVA using Fischer’s LSD post hoc test to find between (i.e., diet) or within group (i.e., thapsigargin/CDN1163). Levene’s statistics also performed to test the homogeneity of variances. LMC data including Ca^2+^ were analyzed using Two-way ANOVA with Bonferroni’s post hoc test (glucose and insulin) as described earlier. Once we find any interaction or main effects, we performed follow-up ANOVA using Fisher’s LSD test. The homogeneity of variance was performed using Levene’s statistics. Statistical analyses were performed using SPSS software (IBM Corp, Armonk, New York, USA). All values are expressed as mean ± SE. The significance level was set at *p* < 0.05. All graphs were generated with Prism 5 (GraphPad Software, La Jolla, CA, USA).

## Data Availability

The datasets analyzed during the current study are available from the corresponding author on reasonable request.
